# Repeated Exposure to D-Amphetamine Decreases Global Protein Synthesis and Regulates the Translation of a Subset of mRNAs in the Striatum

**DOI:** 10.3389/fnmol.2016.00165

**Published:** 2017-01-10

**Authors:** Anne Biever, Jihane Boubaker-Vitre, Laura Cutando, Irene Gracia-Rubio, Mauro Costa-Mattioli, Emma Puighermanal, Emmanuel Valjent

**Affiliations:** ^1^Centre National de la Recherche Scientifique (CNRS), UMR-5203, Institut de Génomique FonctionnelleMontpellier, France; ^2^Institut National de la Santé et de la Recherche Médicale (INSERM), U1191Montpellier, France; ^3^Université de Montpellier, UMR-5203Montpellier, France; ^4^Department of Neuroscience, Memory and Brain Research Center, Baylor College of Medicine, HoustonTX, USA

**Keywords:** D-amphetamine, protein synthesis, striatum, translation factors, eIF2α, Arc/Arg3.1

## Abstract

Repeated psychostimulant exposure induces persistent gene expression modifications that contribute to enduring changes in striatal GABAergic spiny projecting neurons (SPNs). However, it remains unclear whether changes in the control of mRNA translation are required for the establishment of these durable modifications. Here we report that repeated exposure to D-amphetamine decreases global striatal mRNA translation. This effect is paralleled by an enhanced phosphorylation of the translation factors, eIF2α and eEF2, and by the concomitant increased translation of a subset of mRNAs, among which the mRNA encoding for the activity regulated cytoskeleton-associated protein, also known as activity regulated gene 3.1 (Arc/Arg3.1). The enrichment of Arc/Arg3.1 mRNA in the polysomal fraction is accompanied by a robust increase of Arc/Arg3.1 protein levels within the striatum. Immunofluorescence analysis revealed that this increase occurred preferentially in D1R-expressing SPNs localized in striosome compartments. Our results suggest that the decreased global protein synthesis following repeated exposure to D-amphetamine favors the translation of a specific subset of mRNAs in the striatum.

## Introduction

Repetitive behaviors observed following repeated exposure to psychostimulant drugs result in part from imbalanced activity of striatal neural circuits (Canales and Graybiel, 2000). Convergent evidence suggests that long-lasting molecular changes leading to persistent alterations of synaptic properties and spines morphology of striatal projection neurons (SPNs) contribute to the distortion of these circuits (Russo et al., 2010; Luscher and Malenka, 2011). While transcriptional and epigenetic modifications are clearly necessary for the establishment of these persistent changes (Robison and Nestler, 2011), less is known about the ability of repeated psychostimulant exposure to impact on the regulation of protein synthesis through the control of the translational machinery.

Initiation is the rate-limiting step of translation and is tightly controlled by two main mechanisms. The first involves the formation of the eukaryotic initiation factor 4 F (eIF4F) complex, which is regulated by the mechanistic target of rapamycin complex 1 (mTORC1). The second mechanism controls the availability of the ternary complex through the phosphorylation of the α subunit of the eukaryotic initiation factor 2 (eIF2α). Phosphorylated eIF2α decreases general mRNA translation and upregulates the translation of a subset of selective mRNAs containing upstream open reading frames (uORFs) in their 5′ untranslated region (UTR; Buffington et al., 2014). Besides initiation, elongation represents another step of translational control. Phosphorylation of the eukaryotic elongation factor 2 (eEF2) at T56 impairs its binding to the ribosome, thereby decreasing the rate of protein elongation (Nairn et al., 1985; Ryazanov et al., 1988; Price et al., 1991) but also promotes the translation of a subset of mRNAs involved in the control of synaptic plasticity (Scheetz et al., 2000; Park et al., 2008; Verpelli et al., 2010).

By using ribopuromycylation assay, polysome profiling combined with qRT-PCR, and western blot analysis to study the phosphorylation state of translation factors, we investigated the consequence of repeated D-amphetamine exposure on the control of mRNA translation within the striatum.

## Materials and Methods

### Animals

Eight week-old male C57BL/6 were purchased from Charles River Laboratories (France). Male and female *Drd2-eGFP* heterozygous mice (C57BL/6) were generated as described previously (Gong et al., 2003). Animals were housed under standardized conditions with a 12 h light/dark cycle, stable temperature (22 ± 1°C), controlled humidity (55 ± 10%), and food and water *ad libitum*. All experiments were in accordance with the guidelines of the French Agriculture and Forestry Ministry for handling animals and were approved by the local Ethic Committee (D34-172-13).

### Drugs and Treatments

(+)-α-Methylphenethylamine [D-amphetamine (D-amph)] sulfate salt (10 mg/kg) from Sigma–Aldrich (St.-Quentin-Fallavier, France) was dissolved in 0.9% (w/v) NaCl (saline) and injected intraperitoneally (i.p) in a volume of 10 ml/kg, one injection per day during 5 days. Mice were habituated to handling and saline injection for 3 consecutive days before the day of the experiment.

### Western Blot

After pharmacological manipulation, the striatum (including the nucleus accumbens and dorsal striatum) of one hemisphere was extracted as previously described (Puighermanal et al., 2016a), sonicated in 300 μl of 10% sodium dodecyl sulfate (SDS), and boiled at 100°C for 10 min. Protein quantification and western blots were performed as described (Biever et al., 2015). Primary antibodies against p-eIF2α (Ser51) (1:1000; Cell Signaling, #3398), eIF2α (1:1000; Cell Signaling, #5324), p-eEF2 (Thr56) (1:1000; Cell Signaling, #2331), p-p70S6K (Thr389) (1:1000; Cell Signaling, #9234), p-4EBP1 (Thr37/46) (1:500; Cell Signaling, #2855), 4EBP1 (1:500; Cell Signaling, #9644), OPHN1 (1:1000; Cell Signaling, #11939), ATF4 (1:1000; NeuroMab, #75-345), MAP2 (1:2000; Sigma, #M4403) from Sigma, CaMKIIa (1:1000; Millipore, #05-532), puromycin [1:1000; (David et al., 2012)], and β-actin (1:40000; Abcam, #AB6276) were used. The optical density of the relevant immunoreactive bands (or for all the bands for puromycin staining) was quantified after acquisition on a ChemiDoc XRS System (Bio-Rad) controlled by Image Lab software version 3.0 (Bio-Rad).

### Immunofluorescence

After pharmacological manipulation, tissue preparation and immunofluorescence were performed as described (Bertran-Gonzalez et al., 2008). Primary antibodies against GFP (1:1000; Life Technologies, #A10262), Calbindin-D28k (1:1000; Swant, #CB38), and Arc/Arg3.1 (1:500; Santa Cruz Biotechnology, #sc17839) were used.

### Puromycin Incorporation in Whole Striatal Lysates

Puromycin incorporation was performed as described previously (Biever et al., 2015). Briefly, striata were rapidly dissected on an ice-cooled dish and homogenized using 20 up-and-down strokes of a prechilled glass homogenizer with 800 μl of polysomal buffer containing 50 mM Tris pH 7.8, 240 mM KCl, 10 mM MgCl_2_, 250 mM D-sucrose, 2% Triton X-100, 20 μl/ml emetine, 5 mM DTT, 100 U/ml RNasin (Promega), and protease inhibitor cocktail (Roche). Samples were centrifuged for 5 min at 16,000 × *g* at 4°C and supernatant was incubated with 100 μg/ml of puromycin for 10 min at 4°C and then boiled for 10 min at 100°C. Protein concentrations were determined using BCA protein assay (Pierce, Rockford, IL, USA) and samples were stored at -20°C for further western blot analyses.

### Polysome Profiling

The polysome profiling approach was performed as described previously (Biever et al., 2015). RNA from fractions <2 ribosomes (referred to as non-polysomal ‘NP’) and fractions with ≥2 ribosomes (referred to as polysomal ‘P’) was extracted using the TRIZOL (Thermo Fischer) protocol according to the manufacturer’s instructions. The carrier glycoblue was added before RNA precipitation step during the TRIZOL protocol. To remove potential DNA contamination, fractions were treated with DNAse (Ambion) according to the manufacturer’s instruction. RNA integrity was tested using Fragment Analyzer (Advanced Analytical).

### cDNA Synthesis and Quantitative Real-Time PCR

RNA from non-polysomal and polysomal fractions was reverse transcribed to first strand cDNA using the SuperScript^®^ VILO^TM^ cDNA synthesis kit (Invitrogen). Resulting cDNA was used for quantitative real-time PCR (qRT-PCR), using 2X SYBR Green Mix and LC480 Real-Time PCR System (Roche) as described (Puighermanal et al., 2016b). Analysis was performed using LightCycler^®^ 480 Software (Roche). Results are presented as linearized *C*p-values normalized to the stably-expressed genes β-*actin* or *gapdh* and the ΔCP method was used to give the fold change. The primer sequences used in this study are detailed in **Table 1**.

**Table 1:** Sequences of PCR primers.

Markers	PCR primers	
	
	Forward	Reverse
*Ppp1r15a*	CCTTCTATTTACCCGGAGAGAAGCC	GACAGCAAGGAAATGGACTGTGAC
*Ddit3*	CTGGTATGAGGATCTGCAGGAGGTC	GCAGGGTCAAGAGTAGTGAAGGTT
*Ophn1*	CAAACCCCTGGAAACTTTTCG	ATGACAGATGTAAGTGGCGG
*Atf4*	CCAACGTGGTCAAGAGCTCA	TGGCCGGCTATGGATGATGG
*Map2*	GATCAACGGAGAGCTGACCT	CCTTGTGTTGGGCTTCCTTC
*Camk2a*	TTTGAGGAACTGGGAAAGGG	CATGGAGTCGGACGATATTGG
*Arc/Arg3.1*	CTATACCGTTAGCCCCTATGCCATC	CCCAAGACTGATATTGCTGAGCCTC
*Gapdh*	GGAGCGAGACCCCACTAACA	ACATACTCAGCACCGGCCTC
β*-actin*	CGTGAAAAGATGACCCAGATCA	CACAGCCTGGATGGCTACGT


### Statistical Analysis

All statistical analyses were performed using one-way analysis of variance (ANOVA) for multiple comparisons, followed by Bonferroni *post hoc* test. Student *t*-test with equal variances was used for groups of two, when relevant. Statistical significance was determined as *p* < 0.05. Prism 5.0 software was used to perform statistical analyses.

## Results

### Repeated Exposure to D-Amphetamine Reduces Protein Synthesis in the Striatum

**FIGURE 1:**
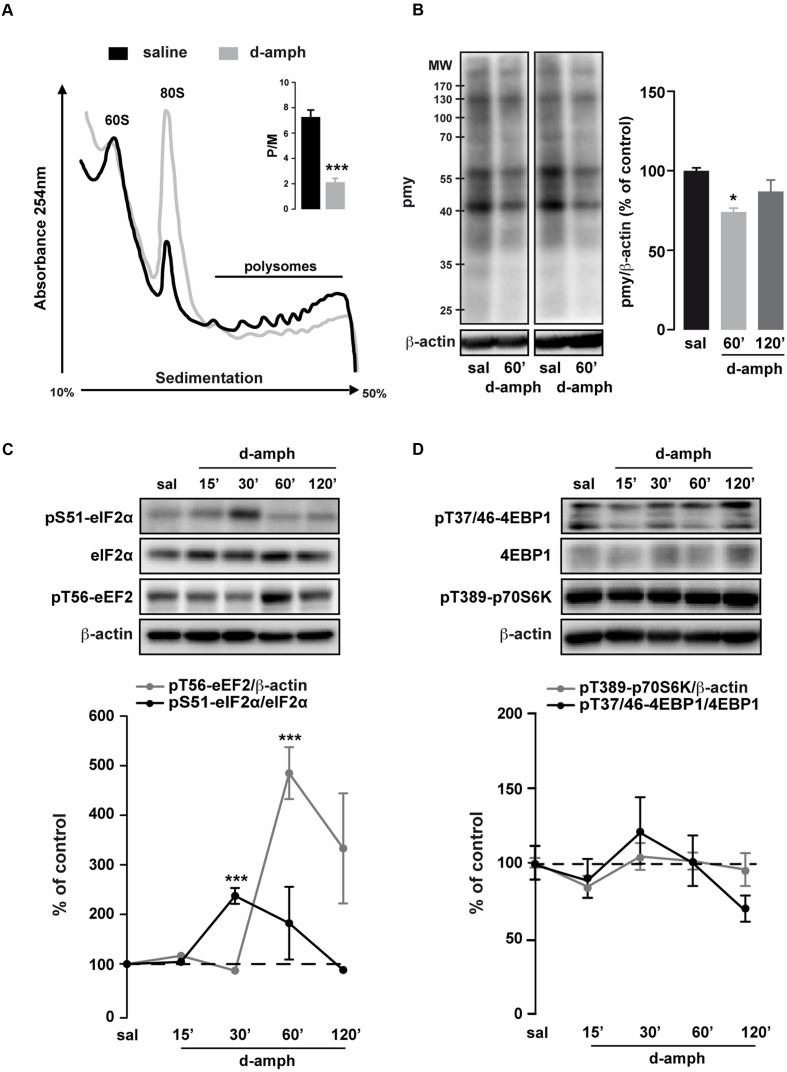
**Repeated D-amphetamine administration decreases global protein synthesis and induces the phosphorylation of the translation factors eIF2α and eEF2.** **(A)** Polysome profiles of whole striatal lysates from mice repeatedly treated with saline or D-amphetamine (10 mg/kg, once daily for 5 days) and killed 60 min after the last injection. Inset: Polysomes (P) to monosome ratio (M) from mice chronically treated with saline or D-amphetamine (mean ± SEM, ^∗∗∗^*p* < 0.001 by unpaired *Student t*-test, *n* = 3 mice/group; right). **(B)** Representative and quantified western blot analysis of puromycin (pmy) staining (normalized to β-actin) of whole striatal lysates incubated 10 min with puromycin from mice killed 60 min after the last injection of saline (sal) or D-amphetamine (D-amph; *n* = 5 mice/group). Results are represented as mean ± SEM. ^∗^*p* < 0.05, by one-way ANOVA followed by Bonferroni *post hoc* test. **(C)** Representative and quantified western blot analysis of phospho-eIF2α (Ser51; normalized to eIF2α) and phospho-eEF2 (Thr56; normalized to β-actin) in striatal lysates of mice treated chronically with saline or D-amphetamine (10 mg/kg, once daily for 5 days) and killed at different time points after injection. **(D)** Phospho-4EBP1 (Thr37/46; normalized to 4EBP1) and phospho-p70S6K (Thr389; normalized to β-actin) similar to **(C)**. Data are expressed as a percentage of saline control (*n* = 5–9 mice/group). Results are represented as mean ± SEM. ^∗∗∗^*p* < 0.001 by one-way ANOVA followed by Bonferroni *post hoc* test.

To investigate whether repeated exposure to D-amphetamine (10 mg/kg, once daily for 5 days) could alter global mRNA translation in the striatum, we performed polysome profile analysis on striatal lysates at 60 min following the last injection of D-amphetamine. We observed an increase in the amplitude of the ‘vacant’ 80S monosome peak along with a reduction in the polysome population in mice treated with D-amphetamine compared to saline-treated mice (**Figure 1A**). Consequently, the polysome to monosome ratio was significantly decreased in D-amphetamine-treated mice (**Figure 1A**, inset).

Polysome profiling is a representation of the steady-state ribosomes engaged in translation. To determine whether repeated exposure to D-amphetamine could modulate *de novo* protein synthesis, we performed an assay adapted from the ribopuromycylation method (David et al., 2012; Biever et al., 2015). We found a transient decrease in puromycin incorporation in striatal lysates of mice treated with D-amphetamine (**Figure 1B**). Altogether, these results indicate that, in the striatum, global mRNA translation was decreased in mice repeatedly exposed to D-amphetamine.

### Repeated Exposure to D-Amphetamine Enhances eIF2α and eEF2 Phosphorylation in the Striatum

The regulation of mRNA translation is tightly controlled by the phosphorylation of translation initiation and elongation factors (Buffington et al., 2014). We therefore analyzed the phosphorylation state of the initiation factor eIF2α and the elongation factor eEF2 in the striatum of mice repeatedly exposed to D-amphetamine. Western blot analysis of whole striatal lysates at different time points (15, 30, 60, and 120 min) following the last D-amphetamine administration revealed a robust enhancement of pS51-eIF2α and pT56-eEF2 at 30 and 60 min post-injection, respectively (**Figure 1C**). The increase in pS51-eIF2α and pT56-eEF2, which was not observed following a single D-amphetamine exposure (**Table 2**), does not result from an accumulation of phosphorylation over the 5 days of the injection protocol as no change was found at earlier time points (e.g., 15 min post-injection for pS51-eIF2α; 15 and 30 min post-injection for pT56-eEF2; **Figure 1C**). The mTORC1 pathway, known to regulate the initiation of translation (Ma and Blenis, 2009), was not activated following repeated D-amphetamine exposure (**Figure 1D**). Together, these results revealed that the decrease in global mRNA translation observed in mice repeatedly administered with D-amphetamine is paralleled by an enhanced eIF2α and eEF2 phosphorylation.

**Table 2:** Effect of acute D-amphetamine administration on the phosphorylation of eIF2α and eEF2.

	Saline	D-amph 15′	D-amph 30′	D-amph 60′
pS51-eIF2α	100 ± 9	134 ± 15	189 ± 34	167 ± 19
pT56-eEF2	100 ± 12	91 ± 11	89 ± 3	105 ± 9


*D-amphetamine (D-amph) was administered at 10 mg/kg and mice were killed at 15, 30, or 60 min post-injection. The level of pS51-eIF2α and pT56-eEF2 was analyzed by western blot and normalized to the unphosphorylated form of eIF2α and β-actin, respectively. Results are represented as mean ± SEM and analyzed by one-way analysis of variance (ANOVA) for multiple comparisons, followed by Bonferroni *post hoc* test.*

### Repeated Exposure to D-Amphetamine Does Not Induce Endoplasmic Reticulum (ER) Stress or Neurotoxicity in the Striatum

Enhanced eIF2α phosphorylation is observed in response to ER stress (Walter and Ron, 2011). To test whether repeated D-amphetamine exposure promoted ER stress in the striatum, the levels of ER stress markers including PERK (PKR-like ER kinase), PDI (protein disulfide isomerase), IRE1α (inositol requiring enzyme 1 α), and BiP (Binding immunoglobulin protein) were analyzed 30, 60, or 120 min following the last D-amphetamine administration. While striatal levels of PERK, PDI, and IRE1α were not altered by D-amphetamine (**Table 3**), BiP levels were transiently decreased only 60 min after the last D-amphetamine administration (**Table 3**).

**Table 3:** Effect of repeated D-amphetamine administration on endoplasmic reticulum stress and neurotoxicity in the striatum.

	Saline	D-amph 15′	D-amph 30′	D-amph 60′
PERK	100 ± 3	113 ± 3	97 ± 2	94 ± 5
PDI	100 ± 4	94 ± 7	90 ± 7	98 ± 10
IRE1α	100 ± 10	93 ± 11	94 ± 12	95 ± 2
BiP	100 ± 6	80 ± 10	47 ± 2^∗∗∗^	82 ± 4
PARP	100 ± 3.7	99 ± 2.7	91 ± 8	96 ± 4
Cleaved Casp3	100 ± 6	95 ± 5	91 ± 9	89 ± 5
Lamin A/C	100 ± 5	103 ± 5	87 ± 9	87 ± 3
GFAP	100 ± 16	118 ± 14	135 ± 44	127 ± 9


*D-amphetamine (D-amph) was administered at 10 mg/kg, for 5 days, one injection per day and mice were killed at 30, 60, or 120 min post-injection. The level of each protein was analyzed by western blot and normalized to β-actin. Results are represented as mean ± SEM and analyzed by one-way analysis of variance (ANOVA) for multiple comparisons, followed by Bonferroni post hoc test, ^∗∗∗^p < 0.001.*

To determine whether repeated D-amphetamine exposure produced apoptosis or gliosis, we analyzed the levels of PARP [Poly (ADP-ribose) polymerase], cleaved caspase 3, cleaved lamin A/C, and GFAP (glial fibrillary acid protein). As summarized in **Table 3**, none of these markers were changed in mice repeatedly treated with D-amphetamine. Moreover, no persistent damage of dopamine terminals was observed as suggested by the stable levels of tyrosine hydroxylase between saline- (100 ± 4) and D-amphetamine-treated (30 min, 95 ± 2; 60 min, 102 ± 16; 120 min, 93 ± 4) mice. Altogether, these results indicate that the increased eIF2α phosphorylation observed in the striatum following repeated D-amphetamine exposure is not accompanied by ER stress or neurotoxicity.

### Enhanced Translation of Selective uORF-Bearing mRNAs by D-Amphetamine

**FIGURE 2:**
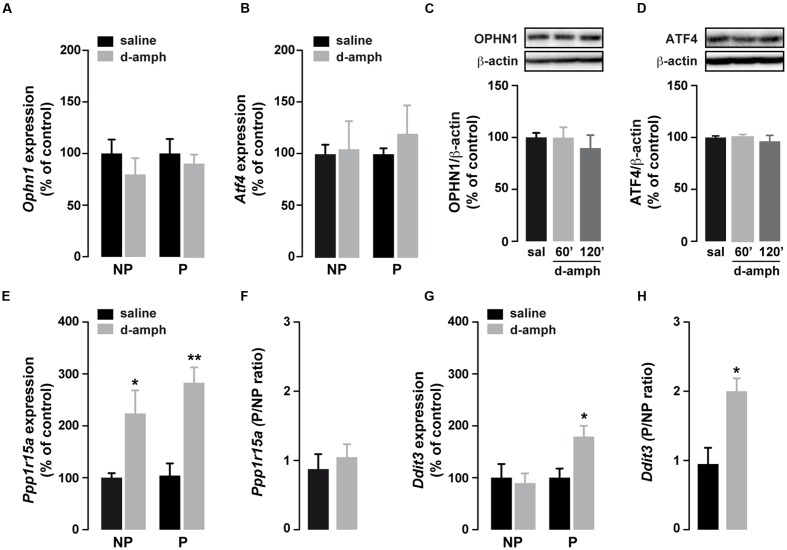
**Repeated d-amphetamine administration increases the translation of a subset of uORF-containing mRNAs.** **(A,B,E,G)** Relative mRNA expression levels of *Ophn1* **(A)**, *Atf4* **(B)**, *Ppp1r15a* **(E)**, and *Ddit3* **(G)** in non-polysomal (NP) and polysomal (P) fractions analyzed by qRT-PCR in the striatum of mice chronically treated with D-amphetamine (10 mg/kg, once daily for 5 days) or saline (*n* = 5 mice/group). All candidate mRNAs were normalized to β-*actin* or *Gapdh* mRNA and expressed as a percentage of saline control. **(C,D)** Representative western blot (top) and quantification (bottom) of OPHN1 **(C)** and ATF4 **(D)** (normalized to β-actin) expression levels in the striatum 60 or 120 min after the last injection of saline (sal) or D-amphetamine (D-amph). Data are expressed as a percentage of saline control (*n* = 5 mice/group). **(F,H)** Ratio of non-polysomal (NP) and polysomal (P) fractions of *Ppp1r15a* **(F)** and *Ddit3* **(H)** mRNAs from the results represented in **(E,G)**, respectively. Results are represented as mean ± SEM. ^∗^*p* < 0.05, ^∗∗^*p* < 0.01 by unpaired *Student t*-test (saline versus D-amphetamine).

Enhanced eIF2α phosphorylation represses global translation but coincidently promotes the translation of mRNAs containing uORFs in their 5′UTRs (Dever, 2002; Buffington et al., 2014). To determine whether repeated exposure to D-amphetamine could increase the translation of specific uORF-bearing mRNAs, we performed polysome profiling combined with qRT-PCR to analyze mRNA levels in the non-polysomal fraction (poorly or not translated mRNAs) and in the polysomal fraction (actively translated mRNAs) in saline and D-amphetamine-treated mice. To correct for the change in the mRNA abundance in the monosomal fraction, mRNA levels in polysomal fractions were systemically normalized to levels in non-polysomal fractions (P/NP ratio). We first analyzed the level of *Atf4* and *Ophn1*, two mRNAs encoding for the activating transcription factor 4 (ATF4) and oligophrenin 1 (OPHN1), respectively, in polysomal fractions from whole striatal lysates. As shown in **Figures 2A,B**, *Atf4* and *Ophn1* mRNAs were not enriched in polysomal fractions suggesting that repeated D-amphetamine exposure did not enhance the translation of *Atf4* and *Ophn1* within the striatum. In line with these results, ATF4 and OPHN1 protein levels, analyzed 60 and 120 min following the last administration of D-amphetamine, remained unchanged (**Figures 2C,D**). The analysis of *Ppp1r15a* and *Ddit3* mRNAs, two other uORF-bearing mRNAs encoding for the protein phosphatase regulatory subunit 15A and the DNA damage-inducible transcript 3, respectively, revealed an enrichment in polysomal fractions of D-amphetamine-treated mice indicating that these two mRNAs were translationally upregulated (**Figures 2E,G**). However, while *Ppp1r15a* mRNA was also enriched in non-polysomal fractions suggesting that *Ppp1r15a* was both transcriptionally and translationally enhanced (**Figure 2F**), *Ddit3* mRNA was only enriched in polysomal fractions (**Figure 2H**). Altogether, these results indicate that repeated D-amphetamine exposure represses global protein synthesis but concomitantly upregulates the translational efficiency of selective uORF-bearing mRNAs.

### Enhanced Striatal Translation of Arc/Arg3.1 mRNA by D-Amphetamine

**FIGURE 3:**
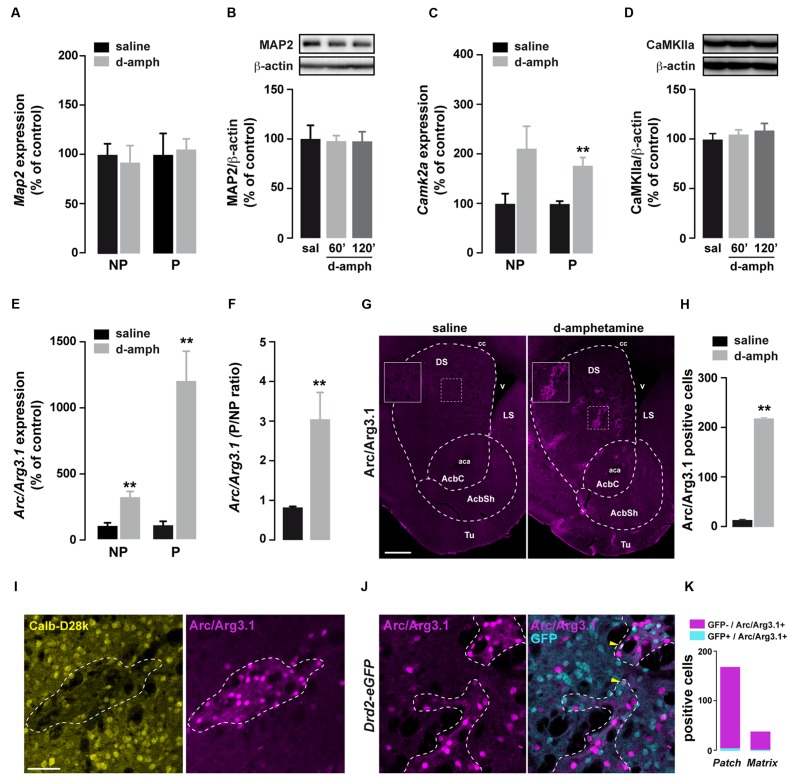
**Repeated D-amphetamine treatment enhances the translation of Arc/Arg3.1.** **(A,C,E)** Relative striatal mRNA expression levels of *Map2* **(A)**, *Camk2a* **(C)**, and *Arc/Arg3.1* **(E)** in non-polysomal (NP) and polysomal (P) fractions analyzed by qRT-PCR 60 min after the last injection of saline or D-amphetamine (10 mg/kg, once daily for 5 days). All candidate mRNAs were normalized to β-*actin* or *Gapdh* mRNA and expressed as a percentage of saline control (*n* = 5 mice/group). **(B,D)** Quantification and representative western blot of MAP2 **(B)** and CaMKIIa **(D)** (normalized to β-actin) of mice killed 60 or 120 min after the last injection of saline (sal) or D-amphetamine (D-amph; *n* = 5 mice/group). Data are expressed as a percentage of saline control. **(F)** Ratio of non-polysomal (NP) and polysomal (P) fractions of *Arc/Arg3.1* mRNA. **(G)** Single immunostaining for Arc/Arg3.1 in coronal sections of the striatum from mice chronically treated with D-amphetamine (10 mg/kg, once daily for 5 days) or saline and killed 60 min after the last injection. Scale bar: 400 μm. **(H)** Quantification of Arc/Arg3.1-positive cells in the striatum 60 min after the last injection of saline or D-amphetamine (D-amph; *n* = 3 mice/group) **(I)** Double immunostaining for Calbindin-D28k (Calb-D28k; yellow) and Arc/Arg3.1 (magenta) in coronal striatal sections of mice chronically treated with D-amphetamine (10 mg/kg, once daily for 5 days) and killed 60 min after the last injection. Scale bar: 60 μm. **(J)** Double immunostaining for GFP (cyan) and Arc/Arg3.1 (magenta) in coronal striatal sections of *Drd2-eGFP* mice chronically treated with D-amphetamine (10 mg/kg, once daily for 5 days) and killed 60 min after the last injection. Note the presence of Arc/Arg3.1 staining in few GFP-positive cells (yellow arrowhead). **(K)** Quantification of Arc/Arg3.1-positive/GFP-positive and Arc/Arg3.1-positive/GFP-negative cells within patch or matrix compartments in the striatum of *Drd2-eGFP* killed at 60 min after the last injection of saline or D-amphetamine (D-amph; *n* = 3 mice/group).

Besides the slowing of elongation, eEF2 phosphorylation at T56 increases the translation of dendrite-localized mRNAs such as *Camk2a* and *Arc/Arg3.1* (Scheetz et al., 2000; Park et al., 2008; Kenney et al., 2016). We therefore tested whether the enhanced eEF2 phosphorylation induced by D-amphetamine was accompanied by increased binding of *Camk2a*, *Map2*, and *Arc/Arg3.1* mRNAs to polysomes. No changes were observed in the abundance of *Map2* mRNA in non-polysomal and polysomal fractions between saline and D-amphetamine-treated mice (**Figure 3A**). In addition, striatal MAP2 protein levels remained unchanged at 60 and 120 min following the last administration of D-amphetamine (**Figure 3B**). The analysis of *Camk2a* mRNAs revealed an enrichment in both non-polysomal and polysomal fractions from mice repeatedly treated with D-amphetamine (**Figure 3C**). However, no significant changes were found in the *Camk2a* P/NP ratio (saline = 1.8 ± 0.58, D-amphetamine = 1.6 ± 0.07; *p* = 0.14) as well as at the protein level (**Figure 3D**). On the other hand, although *Arc/Arg3.*1 mRNAs were also enriched in both non-polysomal and polysomal fractions (**Figure 3E**), we observed a threefold increase in P/NP ratio of *Arc/Arg3.1* mRNA levels (**Figure 3F**). To determine whether the enrichment of *Arc/Arg3.1* mRNAs in the polysomal fractions was accompanied by an increase at the protein level, we monitored Arc/Arg3.1-positive cells 60 min after the last administration of saline or D-amphetamine. Immunofluorescence analysis revealed a robust increase in Arc/Arg3.1 immunoreactivity in the dorsal striatum and the nucleus accumbens corresponding to both Arc/Arg3.1-positive neurons and neuropil (most presumably dendritic processes; **Figures 3G,H**). This increase occurred preferentially in striosomes/patches (identified as calbindin-D28k-poor zones) and only sparsely in the matrix compartment (**Figure 3I**). Finally, double immunofluorescence analysis of Arc/Arg3.1 and GFP in *Drd2-*eGFP mice showed that D-amphetamine evoked Arc/Arg3.1 expression predominantly in GFP-negative neurons, presumably accounting for D1R-expressing neurons (**Figures 3J,K**). Together, our results suggest that repeated D-amphetamine administration enhances *Arc/Arg3.1* mRNA translation preferentially in a subset of striosomal D1R-expressing neurons.

## Discussion

The present study reveals that, in addition to transcriptional and epigenetic modifications, repeated exposure to D-amphetamine can also alter the regulation of translation within the striatum. While global and TOP mRNA translations were not affected following a single D-amphetamine administration (Biever et al., 2015), the use of polysome profiling and ribopuromycylation-based assay revealed that repeated D-amphetamine administration triggers a transient decrease of steady-state ribosomes engaged in translation as well as *de novo* protein synthesis within the striatum. This reduced efficiency of global mRNA translation was accompanied by an enhanced phosphorylation of the translation initiation factor eIF2α and the elongation factor eEF2, known to slow the initiation and the elongation, respectively (Buffington et al., 2014; Kenney et al., 2014). Interestingly, convergent observations indicate that a reduction of initiation most likely account for the decrease in overall protein synthesis observed in mice repeatedly exposed to D-amphetamine. Indeed, unlike the slowing of elongation, which has been associated with increased polysome formation, the attenuation of the initiation is correlated with a reduction of the polysomal population (Hershey et al., 2012). Moreover, while the increase in phosphorylation of eIF2α occurred rapidly (30 min) after D-amphetamine administration, eEF2 phosphorylation was only detectable at 60 min suggesting that this last event may rather contribute to coordinate the rate of elongation with reduced initiation. Further experiments are required to firmly establish that enhanced phosphorylation of eIF2α and/or eEF2 is causally linked to the reduced global protein synthesis observed in the striatum of mice repeatedly administered with D-amphetamine.

Phosphorylation of eIF2α delays the delivery of initiator tRNAs to initiating ribosomes, thereby reducing global protein synthesis (Pavitt et al., 1998; Dever, 1999; Sonenberg and Hinnebusch, 2009). Paradoxically, this phenomenon favors the translation of mRNAs bearing uORFs located at the 5′ leader of the coding sequence (Lu et al., 2004; Vattem and Wek, 2004). Although the mechanisms of enhanced translation remain to be established, we identified two uORF-containing mRNAs whose translation was increased. The first one, *Ddit3*, encodes the transcription factor CHOP (CCAAT/enhancer-binding protein homologous protein; Ubeda et al., 1996). The enrichment of *Ddit3* mRNA only in the polysomal fraction suggests that *Ddit3* mRNAs did not accumulate with the repeated exposure to D-amphetamine. The second uORF-bearing mRNA that was translationally enhanced encodes for the protein phosphatase 1 (PP-1) regulatory subunit 15A also known as GADD34 (growth arrest and DNA damage-inducible protein; Lee et al., 2009). Interestingly, the selective translation of *Ddit3* and *Ppp1r15a* has been previously shown to rely on enhanced eIF2α phosphorylation, allowing scanning ribosomes to bypass inhibitory uORFs in order to translate the main ORFs (Palam et al., 2011; Young et al., 2015, 2016). Thus, D-amphetamine-induced eIF2α phosphorylation may facilitate bypassing the inhibitory uORF within the *Ddit3* and *Ppp1r15a* mRNAs, thereby explaining the increased binding of these mRNAs to polysomes following drug treatment. In contrast to *Ddit3*, *Ppp1r15a* mRNA was also enriched in the non-polysomal fraction suggesting that *Ppp1r15a* mRNA was also regulated at the transcriptional level by repeated D-amphetamine. As *Ppp1r15a* is a transcriptional target of CHOP (Marciniak et al., 2004), increased CHOP levels could promote a transcriptional upregulation of *Ppp1r15a* by D-amphetamine. By interacting with the catalytic subunit of PP-1, GADD34 forms a phosphatase complex involved in eIF2α dephosphorylation (Connor et al., 2001; Novoa et al., 2001). Thus, the increased transcription and translation of GADD34 could contribute to the establishment of a negative feedback regulatory mechanism aiming to reduce the exacerbated eIF2α phosphorylation induced by D-amphetamine. Strikingly, we did not find changes in the translational efficiency of *Atf4* and *Ophn1*, two uORF-bearing mRNAs, which are translated in an eIF2α-dependent fashion in the hippocampus and in the ventral tegmental area (Costa-Mattioli et al., 2005, 2007; Di Prisco et al., 2014; Huang et al., 2016). Several hypotheses could account for these differences. First, an enhanced initiation at these two mRNAs through phosphorylated eIF2α shortly followed by an inhibition of translation elongation by eEF2 phosphorylation could explain why the synthesis of ATF4 and OPHN1 proteins remains unchanged following D-amphetamine treatment. Alternatively, the expression of uORF-lacking splice variants of these genes in the striatum could account for our findings. Indeed, numerous proteins are encoded by mRNAs with diverse 5′UTRs that are generated by alternative splicing and impose different modes of translational regulation (Martineau et al., 2004; Baranick et al., 2008; Al-Fageeh and Smales, 2009; Riley et al., 2010). Finally, depending on the stimuli triggering eIF2α phosphorylation, the brain areas and the cell types where it occurs, different uORF-bearing mRNAs could be translated. Such possibilities remain to be investigated.

Increased eIF2α phosphorylation is observed when unfolded protein response (UPR) is engaged in response to the accumulation of unfolded or misfolded proteins in the lumen of the ER (Walter and Ron, 2011). The expression of GADD34 and CHOP is increased during this process. While GADD34 comprises a negative feedback loop to reverse the translational attenuation mediated by the enhanced eIF2α phosphorylation, the transcription factor CHOP promotes the expression of genes involved in apoptosis (Wang et al., 1998; Zinszner et al., 1998; Novoa et al., 2001). Previous experiments performed in rats indicate that repeated exposure to psychostimulants, including cocaine, D-amphetamine, or methamphetamine, activates the ER stress response, which through the engagement of apoptotic pathways leads to striatal neurotoxicity (Jayanthi et al., 2004, 2009; Krasnova et al., 2005; Beauvais et al., 2011; Go et al., 2016). Our results clearly highlight that the effects induced by repeated exposure to D-amphetamine are radically different in mice. Thus, despite the increase of translation efficiency of *Ddit3* and *Ppp1r15a* mRNAs observed, several evidence suggest that in our condition the ER stress response is not activated. First, the striatal levels of ER stress markers including PERK, PDI, and IRE1α remained unchanged in mice repeatedly administered with D-amphetamine. Second, the translation of the transcription activator of the integrated stress response ATF4 was not enhanced (Harding et al., 2000). Finally, none of the apoptosis or gliosis markers tested were altered suggesting that the regulation of translation by D-amphetamine most likely contributes to persistent modifications altering striatal plasticity rather than representing a protective mechanism to cope with an insult.

Previous studies indicate that eEF2 phosphorylation may promote the translation of a subset of mRNAs among which the dendritic-localized including *Camk2a, Map2*, and *Arc/Arg3.1* (Scheetz et al., 2000; Belelovsky et al., 2005; Park et al., 2008; Kenney et al., 2016). While the translation of *Camk2a* and *Map2* within the striatum was unchanged, we found that D-amphetamine enhances the transcription and translation of Arc/Arg3.1. Indeed, mice repeatedly treated with D-amphetamine displayed a strong enrichment of *Arc/Arg3.1* mRNA in both non-polysomal and polysomal fractions, which was accompanied by an increase of Arc/Arg3.1 protein levels. Although pharmacological or genetic manipulations of eEF2 phosphorylation are required to causally link this event to the D-amphetamine-induced *Arc/Arg3.1* mRNA translation, alternative molecular mechanisms could contribute to the regulation of Arc/Arg3.1 translation. Indeed, enhanced Arc/Arg3.1 synthesis can occur through ERK/Mnk1 signaling and independently of eEF2 phosphorylation in the hippocampus (Panja et al., 2009, 2014). Increased Arc/Arg3.1 levels were preferentially found in the D1R-expressing SPNs located in striosome compartments. These results extend earlier studies showing that acute psychostimulant administration upregulates Arc/Arg3.1 transcripts and protein levels in the striatum (Fosnaugh et al., 1995; Tan et al., 2000; Klebaur et al., 2002; Fumagalli et al., 2006; Salery et al., 2016). While Arc/Arg3.1 expression is often used as a marker of neuronal activity, convergent evidence suggests that the induction of this plasticity-associated gene would sustain homeostatic responses. Thus, by interacting with endophilin 2/3 and dynamin, Arc/Arg3.1 promotes AMPA receptor endocytosis, thereby contributing to synaptic scaling in the hippocampus (Chowdhury et al., 2006; Park et al., 2008). Future studies will be required to determine whether this mechanism could provide a molecular basis for the decreased AMPAR/NMDAR ratio observed in the striatum following repeated exposure to psychostimulants (Thomas et al., 2001; Kourrich et al., 2007; Kasanetz et al., 2010) and could account for the development of increased motor responses induced by D-amphetamine.

## Conclusion

Our study provides evidence that repeated administration of D-amphetamine modulates striatal gene expression not only through the regulation of transcription but also by controlling the translational machinery. Moreover, our results further support the hypothesis that a transient decrease of global mRNA translation efficiently favors the translation of a selective subset of mRNAs (Di Prisco et al., 2014; Huang et al., 2016).

## Author Contributions

AB and EP conducted all the experiments. IG-R and LC helped with biochemical studies. JB-V performed qRT-PCR experiments. AB, EP, and EV designed research. AB and EP analyzed the results. AB, EP, and EV wrote the manuscript with the help of MC-M.

## Conflict of Interest Statement

The authors declare that the research was conducted in the absence of any commercial or financial relationships that could be construed as a potential conflict of interest.
